# Die prekäre alte Normalität der EU und die Notwendigkeit zur Reform

**DOI:** 10.1007/s10273-021-3031-1

**Published:** 2021-10-23

**Authors:** Hubert Gabrisch

**Affiliations:** wiiw, Wiener Institut für Internationale Wirtschaftsvergleiche, Rahlgasse 3, 1060 Wien, Österreich

**Keywords:** F15, F45, H12

## Abstract

Die EU befindet sich seit 2008 im Krisenmodus. Die Sehnsucht nach der alten Normalität ist das große politische Ziel, aber eine Rückkehr zum Status quo ante wäre nicht wirklich wünschenswert. Leider besitzt die europäische Politik kein Konzept für die Zukunft der EU nach der COVID-19-Pandemie. Das könnte sich als existenzielles Problem für die EU in ihrer derzeitigen Gestalt erweisen. Der Artikel analysiert den inneren Zusammenhang der verschiedenen Krisen seit 2008, zeigt Möglichkeiten für eine Reform der Architektur der EU in den zentralen Feldern Fiskal- und Geldpolitik auf, warnt vor weiteren Krisen und vor einer Marginalisierung der EU im globalen Maßstab.

## Abstract (en)

The EU has been in crisis mode since 2008. The old normality is the big political goal, but a return to the status quo ante is not really desirable. Unfortunately, European politics has no concept for the future of the Union after the COVID-19 pandemic. This could prove to be an existential problem for the Union in its current form. The article analyses the inner context of the various crises since 2008, points out possibilities for reforming the Union’s architecture in the central fields of fiscal and monetary policy and warns against further crises and the marginalisation of the Union on a global scale.

Noch richten Politik, Wissenschaft und Medien in der EU ihre volle Aufmerksamkeit auf den unvorhersehbaren Verlauf der COVID-19-Pandemie, aber die Frage nach der Zukunft der EU ist bereits aufgeworfen. Die Bekämpfung der Folgen all der Krisen seit 2008 hat zu fiskalischen und geldpolitischen Ausnahmezuständen geführt. Die Sehnsucht nach „Normalität“ ist unübersehbar, denn es scheint nicht, dass die politische Führung der EU ein Konzept zur Reform der EU-Architektur besäße. Der Aufruf der EU-Kommission (2021) an die breite Öffentlichkeit vom Frühjahr 2021, Reformideen einzureichen (Future of Europe), erscheint eher hilflos, denn ein konsistenter Entwurf ist hier kaum zu erwarten. Was bleibt, ist der bequeme Weg zurück in die Vorkrisennormalität. Dies wäre jedoch genau der falsche Weg, und die Regierungen müssen einsehen, grundlegende Reformen der EU-Architektur auch um den Preis harter Auseinandersetzungen über eine Änderung der EU-Verträge vorzunehmen.

**Dr. Hubert Gabrisch** war langjähriger Abteilungsleiter und Forschungsdirektor des Instituts für Wirtschaftsforschung Halle (IWH) und ist gegenwärtig Senior Research Associate am Vienna Institute for International Economic Studies.

## Ein Unglück kommt selten allein

Die EU hatte nach der Gründung ihrer Währungsunion (EWU) 1999 neun „gute“ Jahre. Der Euro blieb nach innen und nach außen stabil, und er begann, seine erwartete Rolle als eine internationale Reservewährung zu spielen – ein Hinweis auch auf die steigende politische Bedeutung der EU. Zwischen 2004 und 2007 wurden zwölf neue Länder aufgenommen, darunter zehn ehemals sozialistische Länder. Als Mitglied der Welthandelsorganisation (WTO) kann die EU ihr kumuliertes Stimmrecht bei diversen Abkommen (GATT, GATS, TRIPS)[1] ausüben. Die Gründung der EU erschien als gelungener Akt.

Was jedoch in den nächsten 14 Jahren folgte, war eine Abfolge von Krisenepisoden, von denen jede auf ihre Art Entstehung und Schwere der nachfolgenden beeinflusste: Bankenkrise und Große Rezession 2008 bis 2009, Eurokrise 2010 bis 2012, gefolgt von einer kleinen Rezession 2012 bis 2013, Stagnation bis 2019 und dann Ausbruch der Corona-Pandemie Anfang 2020. Trotz temporärer Erholungen nach jedem Tiefpunkt zeichnet sich mittlerweile eine signifikante Wachstumsschwäche der EU im Vergleich mit den wichtigsten Wirtschaftsräumen der Welt ab (vgl. Abbildung 1) – mit deutlichen Desintegrationstendenzen als Folge. Polen, Tschechien und Rumänien lehnen die an sich verpflichtende Übernahme der gemeinsamen Währung ab (Gabrisch und Kämpfe, 2013), Großbritannien verließ die EU 2020.

1 GATT: Allgemeines Zoll- und Handelsabkommen; GATS: Abkommen über den Handel mit Dienstleistungen einschließlich Direktinvestitionen; TRIPS: Übereinkommen über handelsbezogene Aspekte der Rechte des geistigen Eigentums. Diese drei Abkommen formen die WTO.

Abbildung 1

**Reales Bruttoinlandsprodukt der EWU und globaler Konkurrenz**

jährliche Änderung in %

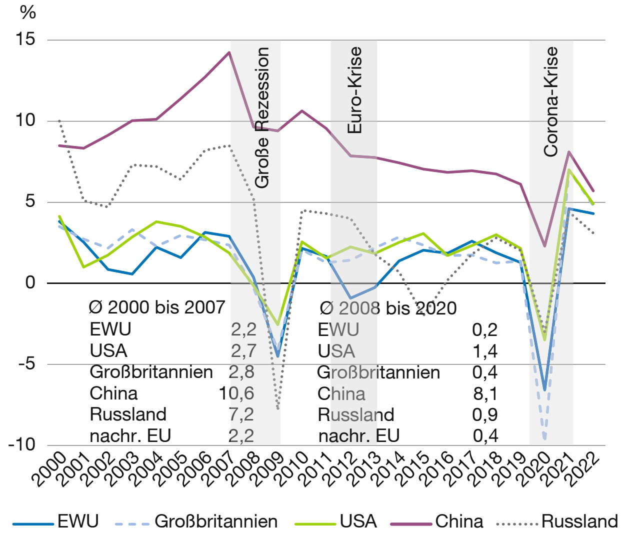

Anmerkungen: EWU = 19 Länder; EU = 27 Länder; China inklusive Hongkong.

Quellen: Eigene Berechnungen nach Daten von Eurostat (1999 bis 2020) und IWF: World Economic Outlook Update Juli 2021 bis 2022.

## Die gar nicht so guten Jahre

Die Anfälligkeit der EU und des Euroraums gegenüber den Finanzmarktschocks der Subprime-Krise kann kaum ohne Blick auf die wirtschaftlichen Spannungen und strukturellen Verwerfungen bereits in der Dekade der scheinbar guten Jahre verstanden werden. Nirgendwo wurde in den Kernländern der Währungsunion ein hohes Wirtschaftswachstum erreicht, das von den 1950er bis zu den 1970er Jahren nahezu Vollbeschäftigung gesichert hatte. Bereits von 1999 bis 2008 wurde – verglichen mit diesen früheren Jahren – nur ein schwaches durchschnittliches Wirtschaftswachstum erzielt (vgl. Abbildung 1).

Unter Ökonom:innen herrscht heute überwiegend Übereinstimmung[2] darin, dass die Krisenanfälligkeit nicht auf die mangelnde Einhaltung des Stabilitäts- und Wachstumspakts zurückzuführen ist (mit Ausnahme vielleicht Griechenlands), sondern auf eine hohe Nettokreditaufnahme des Privatsektors in den peripheren Mitgliedsländern des EWU-Raums (Spanien, Italien, Portugal, Griechenland, Irland, Zypern) und in dessen Kernländern (vor allem Deutschland und Großbritannien). Portfolioumschichtungen von Vermögenden können negative Folgen in Form von Leistungsbilanzdefiziten in den Empfängerländern und Überschüssen in den Herkunftsländern generieren (Saraceno und Tamborini, 2020). In der EWU sind diese Umschichtungen die Folge einer Konvergenz der Anlagerisiken (vor allem Wegfall des Wechselkursrisikos) und der durch die Geldpolitik der EZB hergestellten gleichen Finanzierungsbedingungen (One Size Fits All) zugunsten der EWU-Länder mit hohem Kapitalbedarf. Darüber hinaus wurde im Eurosystem eine regional hohe Kreditvergabe in Verbindung mit ebenso hohen Wirtschaftswachstums- und Inflationsraten als Ausdruck von Konvergenz- und Aufholprozessen vermutet (unter anderem Fischer, 2007).

Auf der realwirtschaftlichen Seite gingen die Finanzströme Hand in Hand mit einer Entkoppelung der nationalen Produktivitätsentwicklung von der Lohnkostenentwicklung in Defizit- wie auch Überschussländern. Gabrisch und Staehr (2014) fanden anhand von Kausalitätstests, dass gewachsene Nettokapitalströme die relativen Lohnstückkosten besser erklären als umgekehrt die Lohnstückkosten die Nettokapitalströme. Baldwin et al. (2015) sahen die Probleme in einer Expansion der Kreditvergabe in den späteren Krisenländern in deren institutionellem Umfeld ohne eigene Geldpolitik. Diese Erklärungen schließen ein zunächst weit verbreitetes Krisennarrativ, das darauf verweist, dass im EWU-Raum die Lohnpolitik nicht an makroökonomischer Stabilisierung orientiert war (unter anderem Flassbeck, 2007; Sinn, 2010; Lapavitsas et al., 2010; Stockhammer, 2011), zwar nicht vollständig aus, relativieren es aber in seiner Bedeutung.

## Episode I: Externer Schock

Die Schockwellen der US-amerikanischen Finanzkrise ab 2007 lösten im EU-Raum eine sinkende Kreditversorgung des Bankensektors aus, der über eine Bilanzkürzung exponierte Risiken zu beseitigen suchte und dabei allgemein zu einem Ausfall von Nachfrage beitrug. Im EWU-Raum waren von den Beschränkungen der Refinanzierung vor allem jene Mitgliedsländer betroffen, die aufgrund ihrer hohen strukturellen Leistungsbilanzdefizite und höheren Inflationsraten (Spanien, Portugal, Irland, Italien, Griechenland) besonders auf einen unverstellten Zugang zu den Finanzmärkten angewiesen waren.

Dieser erste Rückschlag für den Euroraum konnte durch eine temporär abgestimmte expansive Fiskal- und Geldpolitik pariert werden: Die fiskalpolitischen Regeln des Wachstums- und Stabilitätspakts wurden ausgesetzt, um umfangreiche Konjunkturprogramme und Bankenrettungspakete zu erlauben. Allerdings führte die staatliche Bankenrettung zu einer Verschiebung der privaten Überschuldung zum öffentlichen Sektor, insbesondere in den Ländern mit vormaliger exzessiver Nettokreditaufnahme im Eurosystem. Zusätzlich trieb der allgemeine Rückgang der wirtschaftlichen Leistung in allen EU-Ländern mit

2 Anderer Auffassung war unter anderem Wyplosz (2014).

Ausnahme Polens den Staatsschuldenstand der meisten Länder weit über das Maß hinaus, das der Stabilitäts- und Wachstumspakt erlaubte. Die EZB senkte zwar ihre Zinssätze, allerdings zögerlicher als die Zentralbanken in den USA und Großbritannien.

## Episode II: Interner Schock

Die zweite Episode wurde mit der nach 2009 durch die EU-Kommission gemeinsam mit Deutschland und anderen EU-Ländern geforderten Rückkehr zu den Restriktionen des Stabilitäts- und Wachstumspakts eingeleitet. Sie erzwang die Konsolidierung der öffentlichen Haushalte, besonders aber in den Krisenländern. Die EZB folgte zunächst diesem Kurs, indem sie Mitte 2011, als sich die wirtschaftliche Lage im EWU-Raum aufgrund der fiskalischen Restriktionen verschlechterte, ihre Zinssätze wieder erhöhte. Den Eurokrisenländern drohte die Staatsinsolvenz, da es hier zu stärkeren Einbußen bei der Wirtschaftsleistung kam und die Finanzmärkte die Rückzahlungsfähigkeit der öffentlichen Schuldner anzweifelten, den Ausstieg aus dem Euro befürchteten und deshalb Abwertungsrisiken in die Staatsanleihen einpreisten. Die EZB legte zwischen 2010 und 2012 ihr Securities Market Program (SMP) auf, mit dem sie auch Staatsanleihen aus peripheren Mitgliedsländern ankaufte, sterilisierte aber die Transaktionen mit anderen Instrumenten. Nach der wirtschaftlichen Erholung 2010 folgte ein erneuter Einbruch der Wirtschaftsleistung bis 2013 (der Double Dip). Im Fall von Griechenland wurde 2010 das Bailout-Verbot der EU-Verträge durch Darlehen anderer Mitgliedsländer verletzt. Durch die Einsetzung der Troika aus EU-Kommission, IWF und EZB wurden zunächst Griechenland, später Irland und Portugal fiskalische Anpassungsprogramme gegen Kredithilfen aus der 2010 geschaffenen temporären Europäischen Finanzstabilisierungsfazilität auferlegt, die der IWF und später auch die EZB als zu rigide kritisierten. Die Quote der öffentlichen Investitionen am Bruttoinlandsprodukt sank in der gesamten EU und im EWU-Raum, am stärksten jedoch in den Krisenländern und hier insbesondere die Bruttoanlageinvestitionen im Gesundheitssektor (vgl. Abbildung 2a und 2b). Erneute Portfolioumschichtungen erfolgten nun von den Krisenländern in die Kernländer (Flight to Safety). Zypern (2013 bis 2015) und Griechenland (2015 bis 2019) beschränkten zeitweise den freien Kapital- und Geldverkehr. Der Europäische Rat beschloss eine Verschärfung der fiskalischen Regeln 2011 über das sogenannte Six Pack und 2012 über den Fiskalpakt. Gleichwohl: Die Währungsunion war an den Rand ihres Scheiterns geraten.

## Episode III: Stagnation

Die dritte Episode zeichnet sich durch eine offen konträre Stellung von expansiver Geldpolitik der EZB und kontraktiven nationalen Fiskalpolitiken aus, die zu einer nur langsamen Erholung des Wirtschaftswachstums beitrug. Im Juli 2012 kündigte die EZB „unkonventionelle“ Maßnahmen an (Outright Monetary Transactions – OMT), die auch die Möglichkeit des Ankaufs kurzfristiger Staatspapiere einschloss. 2013 erwog sie sogar, die Troika zu verlassen, weil diese mit ihren fiskalischen Anpassungsanforderungen die EZB dazu brachte, die traditionellen Linien zwischen Geld- und Fiskalpolitik zu überschreiten und ihre Unabhängigkeit in Gefahr zu sehen.[3] Die Nullzinslinie beim Hauptrefinanzierungssatz der EZB wurde im März 2016 erreicht. Mit groß angelegten Anleihe-Ankaufsprogrammen (APP), die erstmals auch den mittelbaren Ankauf von Staatsanleihen peripherer Länder mit längerer Laufzeit erlaubten, suchte sie, über die Zinskurve die Marktzinssätze zu drücken und die Nachfrage nach Investitionen in produktive Anlagen anzuregen. Allerdings nutzten große Unternehmen und Banken die Liquiditätszuflüsse eher für Finanzanlagen mit höheren

Abbildung 2

**Öffentliche Investitionen[1] (durchschnittlich pro Jahr) insgesamt und in den Gesundheitssektor**

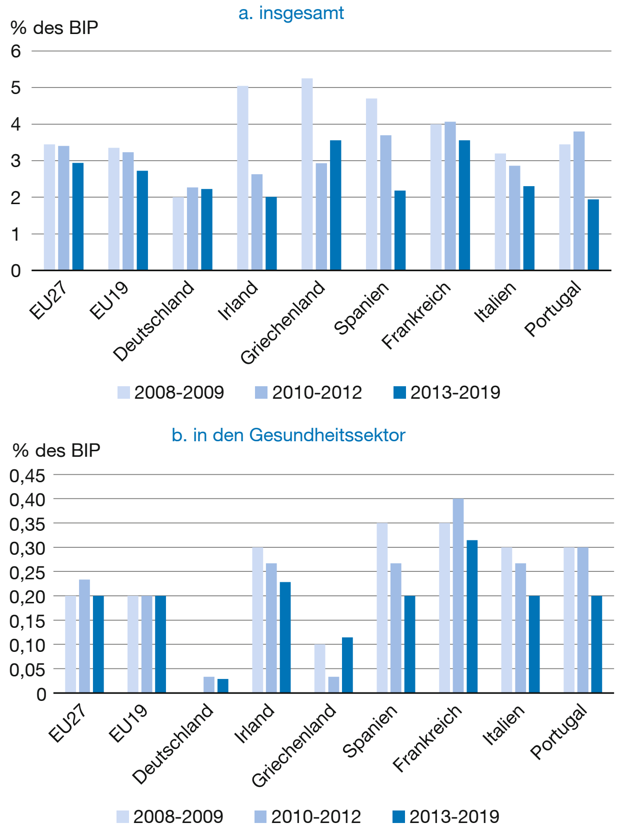

[1] Konsolidierter Staat.

Quelle: Eigene Berechnungen anhand von Eurostat-Daten.

3 Die Troika wurde 2015 mit Hinzutritt des ESM durch die mehr informelle Quadriga ersetzt.

Renditeaussichten (Staatsanleihen und Aktien) bei gesunkenen Risikoprämien als für die Investitionsfinanzierung. Im vierten Quartal 2019 zeigten sich erneut rezessive Tendenzen, die in einzelnen Mitgliedsländern – darunter auch in Deutschland – wieder Forderungen nach Konjunkturprogrammen laut werden ließen.

## Episode IV: Corona-Krise – extern und intern

In dieser bereits von einer erhöhten Krisenanfälligkeit gekennzeichneten Lage brach Anfang 2020 die COVID-19-Pandemie aus. Regional unterschiedliche Ausprägungen der Pandemie wie Todesraten und Intensivbettenbelegungen waren das Ergebnis früherer Kürzungen in der öffentlichen und privaten Krankenvorsorge (vgl. Abbildung 2). Moreno Ibáñez et al. (2021) lieferten mit Querschnittsregressionen für 23 EU-Länder und 44 Regionen in Spanien, das von der Pandemie besonders betroffen war, einen ersten empirischen Einblick in die Zusammenhänge zwischen den Finanzkrisen 2008 bis 2012 und der Schwere der ersten Welle der Pandemie Anfang 2020. Die Autor:innen ermitteln einen signifikanten Zusammenhang zwischen der Zahl der Todesfälle 2020 und dem kumulierten Anstieg der Produktionslücke 2008 bis 2013. Storm (2021) zeigte für 22 OECD-Länder, dass die Unterschiede in den Todesraten auf die unterschiedliche Entwicklung des Gesundheitssystems zurückzuführen sind, insbesondere die öffentlichen Investitionen und Ungleichheiten in der Einkommens-, Vermögensverteilung und dem damit verbundenen Zugang zur Gesundheitsversorgung.

Die wirtschaftlichen Auswirkungen der Pandemie fielen ebenfalls unterschiedlich aus: Reisebeschränkungen trafen die vom Tourismus abhängigen Länder, Krankheitsstände, Lockdowns und Quarantänebestimmungen durchgängig alle Länder. In Ländern, deren Regierung auf die Pandemie nicht mit umfangreichen Ausgabenprogrammen zur Stützung der gesamtwirtschaftlichen Nachfrage reagieren konnte, fiel der Rückgang der Wirtschaftsleistung deutlich stärker aus. Dazu gehörten Griechenland, Spanien, Frankreich, Italien, Österreich und Portugal (alle mit großem Tourismussektor). Der Rückgang ihres realen Bruttoinlandsprodukts lag im ersten Quartal 2021 gegenüber dem Quartal des Jahres 2019 mit dem höchsten Bruttoinlandsprodukt durchgängig bei 16 % und mehr. In Belgien, Deutschland und den Niederlanden lag der Rückgang nur im einstelligen Bereich.[4]

## Nach der Krise ist vor der Krise

In der makroökonomischen Stabilisierungspolitik kam es erneut zu einem Gleichklang von Fiskal- und Geldpolitik,

4 Eigene Berechnungen nach Eurostat-Daten.

Abbildung 3
**Öffentliche Verschuldung[1]**

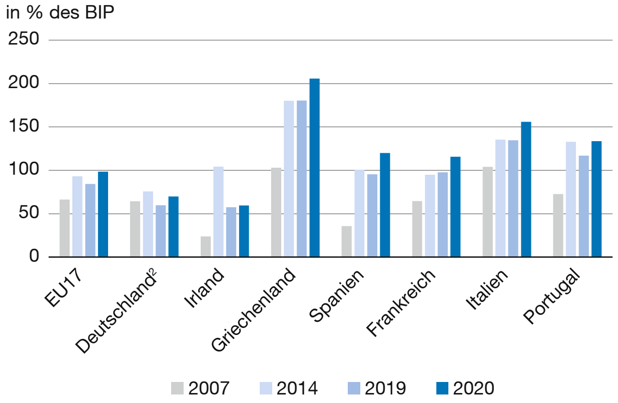

[1] Konsolidierter Staat. [2] bis 1990 ehemaliges Territorium der BRD.

Quelle: Eurostat.

da die Regeln des Stabilitäts- und Wachstumspakts und des Fiskalpakts ausgesetzt wurden. Im ersten Jahr der Pandemie zogen Einkommensausfälle der privaten Haushalte Steuerausfälle und Ausgabenprogramme der öffentlichen Hand einen erneuten Anstieg der Staatsschuldenstände nach sich (vgl. Abbildung 3). Die EZB legte 2020 zusätzlich zu einer Intensivierung ihrer APP ein Pandemie-Notkaufprogramm (PEPP) in Höhe von 1.350 Mrd. Euro auf, deren Mittel größtenteils für den Ankauf nationaler und regionaler Staatsanleihen verwendet werden sollen, darunter erstmals auch griechische (zu den Einzelheiten der Maßnahmen Fendel und Neugebauer, 2021).

Während im August 2021 Geld- und Fiskalpolitik noch an einem Strang ziehen, zeichnet sich in der Fiskalpolitik eine erneute Kurswende ab, da angesichts der zu erwartenden wirtschaftlichen Erholung nach der Pandemie die Rufe nach einer Rückkehr zu den Restriktionen von Stabilitäts- und Wachstumspakt und Fiskalpakt lauter werden (Heine und Herr, 2021, für Deutschland). Die meisten Mitgliedsländer des EWU-Raums weisen mittlerweile Schuldenquoten von mehr als 60 % ihrer Wirtschaftsleistung auf, und der Fiskalpakt fordert, eine etwaige Differenz zwischen der erlaubten Schuldenobergrenze von 60 % der Wirtschaftsleistung und einem höheren Verschuldungsstand jährlich um ein Zwanzigstel zu verringern. Dies wäre jedoch ein Weg mit zweifelhaftem Ausgang, wenn, wie gegenwärtig, eine Produktionslücke besteht, die größer als etwa in den USA ist. Vielmehr ist zu erwarten, dass sich die rezessiven Signale vom Spätherbst 2019 eher verstärken – ähnlich wie 2012 nach Verabschiedung des Fiskalpakts. Abbildung 2 verdeutlicht, dass die öffentlichen Schuldenstände der damaligen Eurokrisenländer Griechenland, Italien, Spanien und Portugal stärker im gesamten EWU-Raum gestiegen sind. Erneute rezessive Tendenzen könnten die Zweifel der Finanzmärkte an der Solvenz des staatlichen Schuldners gerade dieser Länder und an ihrem Verbleib im EWU-Raum und Binnenmarkt wieder wachsen lassen, sodass die EZB ihre bisher eingeschlagene expansive Politik auf unabsehbare Dauer fortsetzen muss. Hinzu käme erwartungsgemäß ein Wiederanstieg notleidender Kredite im Bankensystem, der durch geldpolitische Maßnahmen allein nicht zu bekämpfen ist.

## Das Trilemma der Währungsunion

Lässt man die wirtschaftspolitische Entwicklung Revue passieren, so kann der EZB bescheinigt werden, seit 2012 „geldpolitisch richtig gehandelt“ zu haben (Heine und Herr, 2020, 371). Das zentrale Problem sind die dezentralisierten, allerdings demokratisch legitimierten Fiskalpolitiken, die gegenüber der zentralisierten Geldpolitik einer unabhängigen EZB an Restriktionen gebunden sind und zu einer prozyklischen Ausrichtung neigen. Die Entstehung einer neuen Krise aus der vorhergehenden ist demnach zwar nicht ausschließlich, aber doch auch aus der Rückkehr der Fiskalpolitik zu ihren Beschränkungen nach deren temporärer Suspendierung zu erklären. Es ist wie eine Unwucht, die das EU-Fahrzeug zu einer langsamen Fahrweise zwingt. Bonatti et al. (2020, 16) identifizieren hier ein Trilemma, in das der EWU-Raum spätestens durch die Pandemie gezogen wurde. Auf der einen Seite müsse die Integrität des Binnenmarkts gesichert werden. Auf den beiden anderen Seiten stünden die geldpolitische Orthodoxie der EZB mit ihrem einseitigen Mandat der Inflationsbekämpfung plus des Verbots einer dauerhaften unmittelbaren Staatsfinanzierung und die fiskalische Orthodoxie (vollständige nationale fiskalische Souveränität bei Defizit- und Verschuldungsrestriktionen). In einer Krise könnten nur jeweils zwei der drei Ziele gehalten werden. Die Überwindung des Trilemmas wäre dann von existenzieller Bedeutung für die EU, und deshalb beschreibt es die alternativen Agenden für Reformen.

## Erosion des Binnenmarkts

Legte sich die Politik weiterhin auf die beiden Orthodoxien der Geld- und Fiskalpolitik fest, so würde die Sicherung gleicher Finanzierungsbedingungen durch die EZB und die Anwendung des präventiven und mehr noch des korrektiven Arms des Stabilitäts- und Wachstumspakts sowie des Fiskalpakts die Spannungen und Ungleichgewichte innerhalb des strukturell ungleichen EWU-Raums erneut verstärken. In einer schweren Krise könnte die Integrität des Binnenmarkts nur durch eine weitere Suspendierung der zwei Orthodoxien aufrechterhalten werden. Letztendlich könnte dann das Risiko wieder zunehmen, dass Länder doch aus der Währungs-EU ausscheiden. Mit Blick auf Griechenland wurde dieses Szenario zwischen 2010 und 2015 unter Ökonom:innen intensiv diskutiert (unter anderem Sinn, 2015). Um dies zu verhindern, müsste zumindest eine der beiden Orthodoxien aufgegeben oder zumindest in ihrer Rigidität gemildert werden. An vorderster Stelle würde die Fiskalpolitik stehen, die hauptverantwortlich für die erwähnte Unwucht ist.

## Gleichstellung der Akteure von Fiskal- und Geldpolitik

Eine Reform der Fiskalpolitik sollte darauf abzielen, den Gleichklang von antizyklischer Geld- und Fiskalpolitik in Krisenzeiten zu sichern. Dazu gibt es verschiedene Alternativen. Die wohl einfachste bestünde darin, der EZB als zentraler geldpolitischer Akteurin einen zentralen fiskalischen Akteur zur Seite zu stellen – ein eher ermüdendes Thema nach fast 30 Jahren ergebnisloser Diskussion, aber gleichwohl von bleibender Aktualität. Das im Juli 2020 als Antwort auf die Pandemie verabschiedete Next Generation EU Package im Umfang von 750 Mrd. Euro, finanziert aus Kreditaufnahmen der EU-Kommission, ist realiter eine erste Schwächung der fiskalischen Orthodoxie, auch wenn der Fonds nur auf drei Jahre angelegt ist. Zielführend wäre dagegen die Einrichtung eines ständig verfügbaren Stabilisierungsmechanismus. Die Vorschläge für ein EU-Finanzministerium oder einen Stabilisierungsfonds, finanziert durch eigene Steuereinnahmen der EU-Kommission oder Kreditaufnahme, liegen seit langem auf dem Tisch.[5] Es liegt auf der Hand, dass die Präsenz eines derartigen Akteurs die disziplinierenden Regeln für die Budgetpolitik der Nationalstaaten nicht aufheben muss, sondern erst sinnvoll und glaubhaft machen kann, wie etwa das Verbot von Bailouts durch andere EU-Länder. Über den Mechanismus könnten EU-Mittel zur Stützung der Investitionen dann mobilisiert werden, wenn nationale Regierungen wie die der Eurokrisenländer in ihren Budgets zunächst und vor allem die öffentlichen Investitionen kürzen.

Eine Änderung der EU-Verträge könnte dadurch umgangen werden, dass ein solcher ständiger Mechanismus auch außerhalb der EU-Verträge eingerichtet werden kann, so wie bereits der EFSM und sein Nachfolger ESM oder der geplante Europäische Währungsfonds. Um einen echten fiskalischen Akteur zu installieren, wären jedoch noch zusätzliche Reformen notwendig, die ohne Änderung der Verträge wohl nicht möglich wären, wie etwa die Aufgabe des Einstimmigkeitsprinzips bei der mehrjährigen Finanzplanung, alleiniges Budgetrecht des EU-Parlaments bei der jährlichen Haushaltsaufstellung und ein geteiltes Budgetrecht nur noch bei Aufgaben, die aus Zuführungen der Länder finanziert werden.

5 Siehe die intensive Diskussion in Intereconomics (2017) oder auch Bibow (2015).

## Überwindung der geldpolitischen Orthodoxie

Sollte es zu keiner grundlegenden fiskalischen Reform kommen, rückt die EZB in den Fokus. Vor 2012 hatte sie mit dem Ziel, einheitliche Finanzierungsbedingungen im EWU-Raum herzustellen zweifellos keinen Beitrag zur Krisenprävention und -überwindung geleistet und musste danach, als ihr Hauptrefinanzierungssatz an die Nullprozentgrenze stieß, mit unkonventionellen Maßnahmen der restriktiven Fiskalpolitik entgegenwirken, insbesondere dem Quantitative Easing (QE) und neuen Kommunikationsinstrumenten (insbesondere Forward Guidance). Seitdem findet im Umfeld der EZB wie auch anderer Zentralbanken eine Diskussion über Änderungen in den Zielen und Instrumenten der Geldpolitik statt.

Was die Ziele betrifft, zeigen die Erfahrungen, dass anhaltende Preisstabilität keine Garantie für Finanzstabilität ist. Der Schluss liegt nahe, das Ziel der Preisstabilität durch die Finanzmarktstabilität, definiert als Abwesenheit einer Kreditblase und von Vermögenspreisinflation, zu ergänzen. Das mit dieser Erweiterung des EZB-Mandats verbundene Problem ist allerdings, dass – wie zuletzt – bei zu niedriger Inflation entsprechend niedrige Leitzinsen einen Vermögenspreisboom begünstigen, der zum Nukleus der nächsten Krise werden kann. Dieser Konflikt kann nur durch eine stärkere Regulierung (Mindestreserven, Kapitalpuffer) des Verbriefungsgewerbes bzw. des Schattenbanksektors gelöst werden.

Zwar hat die Übertragung der Bankenaufsicht über größere Banken 2016 in Verbindung mit Asset Quality Reviews (AQR) und Stresstests das präventive Instrumentarium der EZB erweitert. Aber deren Effektivität wird durch die Existenz eines großen, durch die Zentralbank wenig kontrollierten Schattenbankensystems beschränkt, der für extreme Ausschläge auf den Vermögensmärkten verantwortlich ist. Mit der von der EU-Kommission vorangetriebenen Kapitalmarktunion wird die Aufgabe der EZB nicht leichter, da über die Förderung des grenzüberschreitenden Handels mit Verbriefungen der Kreditzyklus kaum zu glätten ist und die Ansteckungsgefahr in wachsenden Netzwerkzusammenhängen vergrößert wird (Battiston et al., 2012; Gabrisch, 2016).

Was die unkonventionellen Maßnahmen betrifft, fanden Blinder et al. (2017) in einer Umfrage unter Zentralbankangestellten und Forschenden, dass eine deutliche Mehrheit ihren Verbleib im Instrumentenkoffer der Zentralbanken erwartet. Mit dem verstärkten Einsatz von Offenmarktoperationen im staatlichen Anleihesegment ist unter Ökonom:innen auch eine Diskussion über die Möglichkeiten und Grenzen einer Zentralbank als Lender of Last Resort (LOLR) auch für Regierungen ausgelöst worden (Bibow, 2015; BIS, 2014; Tucker, 2014; CEPR, 2018; Bonatti et al., 2021). Diese Diskussion ist erforderlich, weil die Regierungen durch ihre Bankenrettungsprogramme die Rolle eines Rückversicherers für den Bankensektor übernommen hatten. Die EZB selbst ist mit ihren Anleiheankaufsprogrammen zum Rückversicherer für Regierungen und damit natürlich mittelbar auch für den Bankensektor geworden. Warum dann nicht eine unmittelbare LOLR-Rolle in der Bankenrettung, die nach traditionellem Verständnis der Zentralbank zufällt – nach den Bagehot-Standards? Dazu müsste das Diskontfenster der EZB (Spitzenrefinanzierungsgeschäft) oder der nationalen Zentralbanken (Emergency Liquidity Assistance – ELA) ausgeweitet werden. Die üblichen Einwände – Moral Hazard und die Unterscheidung von Insolvenz und Illiquidität – relativieren sich, da eine erweiterte LOLR-Funktion der EZB das „größte Potenzial" (Bibow, 2015) besitzt, eine schwere Krise zu verhindern, wenn die Fiskalpolitik ausfällt.

## Die drohende globale Marginalisierung der EU

Ein Verzicht auf Reformen und eine Rückkehr zur „Normalität" hätte neben ökonomischen auch einige politische Konsequenzen. Zunächst einmal würde die Fähigkeit der EU geschwächt werden, zu einer wichtigen globalen Akteurin zu werden, vor allem in der Systemrivalität mit China. Dazu ist noch einmal ein Blick auf Abbildung 1 aufschlussreich, welche die hohe Dynamik der chinesischen Wirtschaft zeigt. Branko Milanović (2020, 297-302),[6] hat ausgerechnet, dass es nur noch eine Generation (ca. 24 Jahre) dauern dürfte, bis bei unverändertem Wachstumsdifferential der gegenwärtig bestehende Unterschied in den durchschnittlichen EU-weiten Pro-Kopf-Einkommen zu China nivelliert wäre. In den Augen von Autokratien waren Demokratie und Rechtsstaat schon immer veraltete Staatsmodelle, die breiten Bevölkerungsschichten kein hohes Wirtschaftswachstum und wachsenden Wohlstand zu bieten vermögen. Heute versucht insbesondere das nach globaler Dominanz strebende China dieses Bild in Afrika, im asiatisch-pazifischen Raum, aber auch in Mittel- und Osteuropa zu verbreiten. Die EU kann nur dann ein Modell für andere Weltregionen sein, wenn sie selbst als wachsender Absatzmarkt für diese Länder attraktiv ist. Gegenwärtig verlieren ihre am westlichen liberalen Modell orientierten Wertvorstellungen in Asien und Afrika nicht nur aber auch angesichts der schwachen Dynamik ihres Binnenmarkts an Überzeugungskraft. Auf lange Frist würde die EU die Systemauseinandersetzung zwischen liberaler Demokratie und autoritär-totalitärem Staatsverständnis verlieren.

6 Bei einem durchschnittlichen Wachstum des BIP pro Kopf in Kaufkraftparitäten von 2 % in der EU und 6 % in China.

Was die Attraktivität der EU nach innen betrifft: Liberale Demokratien ziehen einen Großteil ihrer inneren Legitimität daraus, dass sie eine ständige Verbesserung der Lebensumstände bieten müssen. In nicht kleinen Bevölkerungssegmenten der westlichen Länder, einschließlich der EU, nehmen demokratische Teilhabe und Akzeptanz individueller Rechte einen geringeren Stellenwert ein als eine ständig verbesserte Versorgung mit Konsumgütern und Wohnung. Der Aufstieg politischer Bewegungen mit autoritärer Ausrichtung in Deutschland und anderen EU-Staaten wird sich fortsetzen, wenn autoritäre oder „illiberale" Demokratiemodelle (z. B. in Polen und Ungarn) beweisen, dass sie diese Erwartungen besser erfüllen können. Es ist also an der Zeit, Konsequenzen zu ziehen und die Unwucht in der europäischen Wirtschaftspolitik zu beseitigen.

**Title:** ***The EU's Precarious Old Normal and the Need for Reform***

**JEL Classification:** *F15, F45, H12*
